# 

**Published:** 1812-11

**Authors:** 


### MONTHLY CATALOGUE OF MEDICAL BOOKS.

CONSPECTUS Medicinæ Theoreticæ ad Usum Academicum, Auctore Jacobo Gregory, M. D. Olim Med. Theor. nunc Med. Pract. in Acad. Edin. Prof. Editio Quarta Prioribus Auctior et Emendatior. One large Volume 8vo.—Highley.

A Grammar of Medicine, exhibiting the General Elements of Medical Science, in a popular and compressed form, for the use of Pupils and Students, and of Young Persons in Public Schools, who are destined for any department of the Medical Profession.

A Treatise on the Blood, Inflammation, and Gun-shot Wounds. By the late John Hunter. Two Vols. 8vo. A new Edition.—Cox.

Anatomical Examinations—a complete Series of Anatomical Questions with Answers. The Answers arranged so as to form an elementary System of Anatomy, and intended as preparatory to Examinations at Surgeons' Hall. To which is annexed, Tables of the Bones, Muscles, and Arteries. The Second Edition, corrected. Two Volumes, small 8vo.—Highley.

Nosology, or a Systematic Arrangement of Diseases into Classes, Orders, Genera, and Species, with accurate Definitions. Translated from the Latin of William Cullen, M.D. Small 8vo.—Highley.

The Edinburgh New Dispensatory, containing the Natural, Chemical, and Medical, History of the Substances admitted into the Materia Medica; including the Pharmaceutical Preparations and Compounds of the latest London, Dublin, and Edinburgh, Pharmacopœias. Arranged upon a new Plan. By John Thomson, M.D. The Pharmaceutical Preparations and Compounds of each Article in the Materia Medica are collected under one simple distinct Head. One thick Volume 8vo.—Highley.

Observations on the Nature and Treatment of Tinea Capitis, or Scald Head; and on Impaired Vision arising from Opacity of the Cornea. By Thomas Luxmoore. 8vo.—Highley.

Practical Observations on Strictures of the Urethra; with Cases illustrative of the comparative Merits of the Caustic and Common Bougie. To which is now subjoined, an Appendix, containing an improved Method of treating Urethral Complaints by the Employment of a new Instrument, as well as by the Catheter. By Thomas Luxmoore. One Volume, 8vo.—Highley.

Recent

### *Recent American Publications.*

The General Repository and Review, to be continued quarterly. Nos. I. and II. containing 474 pages. Cambridge; Hilliard, 1812.

A Treatise on Bridge Architecture, in which the superior Advantages of the Flying Pendant Lever Bridge are fully proved; with an Historical Account and Description of different Bridges erected in different Parts of the World, from an early Period down to the present Time. By Thomas Pope, Architect, &c. New York; Niven, 8vo.

Communications of the Medical Society of Connecticut. Vol. I. No. 1. New Haven; 8vo.

American Ornithology, or the Natural History of the Birds of the United States; illustrated with Plates, engraved and colored from original Drawings, taken from Nature. By Alex. Wilson. The 4th Vol. 4to.

Two Lectures on Comets, by Professor Winthrop; also an Essay on Comets, by A. Oliver, jun. esq. With Sketches of the Lives of Professor Winthrop and Mr. Oliver. Likewise, a Supplement, relative to the present Comet of 1811. Boston; T. B. Wait and Co. 12mo.

Anniversary Address to the Medical Society of the State of New York; by the President, Dr. Romayne.

Dissertation on the Use and Abuse of Tobacco; wherein the Advantages and Disadvantages attending the Consumption of that entertaining Weed, are particularly considered. Humbly addressed to all Tobacco Consumers, but especially those among Religious People. Second American Edition. By Adam Clarke, LL.D. Newburyport; Thomas and Whipple. 12mo.

Bell's Operative Surgery is in the press. Hale and Hormer, Hertford, Con.

---

### *NOTICES TO CORRESPONDENTS.*

*Mr. James Barlow, of Blackburn, solicits from some of our Correspondents answers to the following Queries:*

1. *What is the earliest period of* GESTATION *for a Child's being born alive so as to be reared?*

2. *What is the latest and most protracted period of* GESTATION *for a Woman bringing forth a living Child?*

3. *What is the usual period of* GESTATION *in Cows, and how far may parturition be protracted in them and other quadrupeds?*

*Another Correspondent requests answers to the following Queries:*

1. *Can an Apothecary distil Æthers, Spirits, &c. for the purpose of dispensing medicines in his own shop, without incurring the penalty of the Excise?*

2. *What is the best Menstruum for dissolving the Gum Copal, and does it require heat for its solution?*

*Communications are received from Mr. Jukes, with a drawing; from Doctors D. J. W. Dickson; Kinglake; T. C. Speer; R. W. Bamfield, esq.; W. P.; &c. &c.*

*We have received the Second Number of the New-England Journal of Medicine and Surgery; and shall shortly present our readers with an account of its contents.*

*Our Irish Friends are respectfully informed, that we have appointed* Mr. M'KEENE, *of Dublin, our Agent for the Sale and Circulation of this Journal in Ireland.*

